# 

**Published:** 1892-03

**Authors:** 


### TABLE OF CONTENTS.

ORIGINAL COMMUNICATIONS. PAGE

The Syrup of Iron Chloride. By G. W. WELD, M.D., D.D.S., New York . . . 161
"We Specialists." By RICHARDSON GREY, M.D., East Orange, N. J. . . . 177
A Consideration of some of the Responsibilities of Dentists and Physicians. By LYMAN F. BIGELOW, D.M.D. . . . 184
Bichloride of Mercury in Dental Practice. By DR. GEORGE S. ALLAN, New York . . . 190
Hydrogen Peroxide in Dental Operations. By DR. D. D. PEABODY, Stoneham, Mass. . . . 192
The Use of Gutta-Percha as a Root-Canal Filling. By FOREST G. EDDY, D.M.D. . . . 195
Pyorrhœa Alveolaris. By DR. EDGAR F. STEVENS, Boston, Mass. . . . 198
Labial Rubber-Dam Clamp. By WILLIAM A. WOODWARD, D.D.S., New York . . . 200
Methods of filling Teeth Forty Years Ago. By FREDERICK N. SEABURY, D.D.S., Providence, R. I. . . . 201

REPORTS OF SOCIETY MEETINGS.

American Dental Association . . . 203
American Academy of Dental Science . . . 216
Odontological Society of Pennsylvania . . . 219
Central Dental Association of Northern New Jersey . . . 226

EDITORIAL.

A Novelty in Treatment . . . 230
Annual Meeting of the International Dental Publication Company . . . 233
First District Dental Society of New York . . . 234
Michigan State Board of Examiners . . . 235
Mass-Meeting of Dentists in New York City . . . 235

OBITUARY.

Dr. Charles P. Pengra . . . 235

BIBLIOGRAPHY . . . 236

CURRENT NEWS.

Michigan State Board of Examiners . . . 238
St. Louis Dental Society . . . 238
Mississippi Valley Association of Dental Surgeons . . . 239
The Post-Graduate Dental Association of the United States . . . 239
Oral Surgery in New York . . . 239

SELECTIONS.

Rudolph Virchow . . . 240

